# Characterization of Odorous and Potentially Harmful Substances in Artists' Acrylic Paint

**DOI:** 10.3389/fpubh.2018.00350

**Published:** 2018-11-29

**Authors:** Patrick Bauer, Andrea Buettner

**Affiliations:** ^1^Chair of Aroma and Smell Research, Department of Chemistry and Pharmacy, Emil Fischer Center, Friedrich-Alexander-Universität Erlangen-Nürnberg, Erlangen, Germany; ^2^Department Sensory Analytics, Fraunhofer Institute for Process Engineering and Packaging IVV, Freising, Germany

**Keywords:** acrylic paints, off-odors, gas chromatography-olfactometry (GC-O), odor extract dilution analysis (OEDA), harmful substances, naphthalene, butyl acrylate, 3-methyl-4-heptanone

## Abstract

Acrylic paints are fast drying water based paints that are easy to handle and have a high covering capacity and therefore possess many characteristics that make them applicable in a wide range of applications, such as varnishes or artists paints. Due to their emitted volatile organic compounds, these paints are associated with different work-related diseases and are known to emit an unpleasant odor. In this study six acrylic paints for artists were analyzed regarding their odor-active constituents. Therefore, the samples were extracted with dichloromethane and purified via solvent assisted flavor evaporation prior to analysis of the distillates by gas chromatography-mass spectrometry (GC-MS), gas chromatography-olfactometry (GC-O), and GC-GC-MS/O. Additionally all samples were sensorially characterized by a trained sensory panel. The identified odorous substances were primarily benzene derivatives (styrene, ethylbenzene, allylbenzene, propylbenzene) with a plastic-like, aromatic and solvent-like odor. Thereby, polycyclic aromatic hydrocarbons (naphthalene, indane, and tetralin derivatives) contributed to the plastic- and mothball-like odor whereas acrylic monomers (butyl acrylate) were found to be responsible for a mushroom-like and geranium leaf-like odor. As most of these substances are also known to be harmful, a reduction or replacement of these substances by less toxic and non-odor active ingredients is likely to turn out to be advisable in order to reduce the odor and potential negative physiological effects of paints.

## Introduction

The historical development of acrylic paint dates back to the early Nineteenth century, when the first acrylic resin dispersion was developed by BASF. Ever since, the composition of acrylic paints changed in various ways to improve the quality as well as safety of the products, and to reduce their toxicity. The introduction of waterborne acrylic paints brought progress in reducing harmful solvents and thus the emission of volatile organic compounds (VOCs). As nowadays water is mostly used as solvent for acrylic paints (cf. Table 1) and the paints usually contain only small amounts of organic solvents, they are commonly recognized as safe by the customer, even though an exposure to VOCs cannot be fully excluded.

**Table 1:** Composition of acrylic paints according to suppliers' information.

**Ingredient**	**Percentage (%)**
Water	10–50
Pigments and extenders	25–50
Acrylic polymer (solution)	20–60
Biocides	<1.0
Additives	10–20

Their fast drying process, the easy handling and their high covering capacity make them suitable not only as artist paints, but also for the application as in- and out-door construction paint, varnishes and other coatings. Painters, artists, and other workers that are exposed to emissions that are released from paints have been, however, reported to suffer from physical harm caused by the emitted VOCs. Whereas, hobby painters sometimes show allergic-like symptoms, feel dizzy or suffer, at times, from headache after long painting sessions, professional painters showed different types of long-term effects depending on the duration and extent of their exposure. Previous studies demonstrated that the emitted VOCs caused stronger asthmatic symptoms in affected persons when being asked to apply acrylic paint on a board for a period of 60 min (1). Likewise, VOCs from acrylic paints were proposed as a cause for occupational asthma (2). The volatile fraction of water-based paints is composed of alcohols, esters or mineral spirits, i.e., a mixture of various long- and branched-chain alkanes (C_8_–C_14_) and volatile aromatic compounds. The latter substances have been reported to be harmful, as hepatic damage and neurotoxic effects have been observed for these volatiles in the past (3). It is important to mention that some of these substances show none or only a low odor activity. Therefore, these substances bare the risk of causing adverse effects on human health after acute or chronical exposure without being recognized by the customer. However, many manufacturers keep on using mineral spirits, as it is an easy and cheap way to attain the desired properties. Besides the influence on human health, acrylic paints have been reported to emit an unpleasant and long lasting odor (4, 5). Despite these observations, there has been barely any research on the odor of paints, and little is known about the causative odor active compounds. In a review article about water based paint, solvents, co-solvents, acrylic monomers and amines have been proposed as potential odor-active constituents of paints (6). Furthermore, the coalescing agent 2,2,4-trimethyl-1,3-pentandiolmonoisobutyrate, also referred to as Texanol (registered trademark of the Eastman Chemical Company), as well as different aromatic hydrocarbons have been reported to contribute to the odor of water based construction paints (5).

To address this lack of knowledge, we aimed to identify the components responsible for the intense odor of acrylic paints. To achieve this goal, the VOCs of six different acrylic paints for artists were analyzed using a dichloromethane (DCM)-based solvent extraction combined with gas chromatography-mass spectrometry (GC-MS), gas chromatography-olfactometry (GC-O) and heart-cut two-dimensional gas chromatography-mass spectrometry/olfactometry (2D-GC-MS/O), representing state-of-the-art analytical methods in odor analysis. The odorants with the highest impact on the odor of fresh acrylic paint were determined by an odor extract dilution analysis (OEDA) and a sensory evaluation conducted by a trained panel was carried out to compare the analytical results with the overall olfactory impression of the paints. The evaluation of the obtained data was used to determine the ingredients that contributed with highest impact to the unpleasant odor of fresh paint. Furthermore, we identified the most prominent non-odorous VOCs in our samples by GC-MS methods. Our goal was, accordingly, to provide the chemical basis that may help manufacturers to develop targeted avoidance strategies for off-odorants and potentially harmful constituents in acrylic paints, and to provide olfactory acceptable and safer products.

## Materials and methods

### Chemicals

Dichloromethane and anhydrous sodium sulfate were purchased from VWR (Darmstadt, Germany). All chemicals were at least of analytical grade. To improve its purity, DCM was freshly distilled prior to use. Acetic acid, benzaldehyde, sec-butylbenzene, 1-butanol, (*E*)-2-butenal, butyl acrylate, decanal, ethenylbenzene (styrene), 2-ethylhexyl acrylate, methyl octanoate, 1-methylnaphthalene, 2-methylnaphthalene, naphthalene, octanal and propylbenzene were obtained from Sigma-Aldrich (Steinheim, Germany). Butyl acetate, ethylbenzene, (propan-2-yl)benzene (cumene) and 3-phenyl-1-propene (allylbenzene), were purchased from TCI Europe (Zwijndrecht, Belgium). 1,2-Dimethylnaphthalene, 1,7-dimethylnaphthalene and 3-methyl-4-heptanone were obtained from abcr (Karlsruhe, Germany), and 2-acetyl-1-pyrroline from aromaLAB (Planegg, Germany).

### Samples

The odor-active substances in acrylic paints were identified in six different samples. The investigated paints were obtained from three different manufacturers (AP1-3), each providing one white and one black paint containing either the pigment titanium white (TW) or carbon black (CB). The black pigment in AP1 samples was further specified as Lamp Black. The paints were commercially available in an online shop and were chosen because they represent different market segments. AP1 samples were provided in a low-cost starter pack and were chosen to represent the exposure of beginners. AP2 samples were among the best-sold paints that were available in the online shop and therefore represent the average exposure of artists that work with acrylic paints. AP3 samples were chosen by the authors because the paints were specified as solvent-free and low in VOC content representing a growing market of toxicological safer products in the paint sector. The closed samples were stored at room temperature for a maximum of 1 month until sample workup.

### Determination of the odor profile

Each sample (2 ml) was presented to the panelists in 10 ml brown glass bottles. The sensory evaluation of the samples was started by separately smelling at the samples followed by the determination of odor qualities that had to be rated in a second sensory test. If an odor quality was named by at least 50% of the panelists, it was selected for quantitative rating in the second test. In the second session, the panelists were asked to rate the intensity of the determined odor qualities on a scale from 0 (no perception) to 10 (strong perception) with 0.5 intermediate steps allowed. Furthermore, the panelists were asked to rate the hedonics, their subjective feeling about possible health hazards and the overall smell intensity on a scale from 0 (dislike, no concern about health hazard and no perception, respectively) to 10. Intermediate steps of 0.5 were allowed. The panel consisted of 12 people (4 male, 8 female) with an age range of 23–55 (median: 25.5). The panelists were trained for at least 6 month in weekly sensory sessions to orthonasally recognize odorants and describe them according to an in-house sensory language that is based on more than 150 odorants that are used for training.

### Samples preparation

For the solvent extraction of volatile compounds 2.5 g paint were mixed with 20 ml dist. water and stirred at room temperature until the two components were thoroughly mixed. After adding 50 ml DCM, the mixture was stirred for 30 min under the same conditions. Then, phases were separated and the aqueous phase was washed twice with approximately 25 ml of DCM in each case, resulting in a total volume of 50 ml. The combined DCM phases underwent a high vacuum distillation using the solvent assisted flavor evaporation (SAFE) technique at 50°C (7). The obtained distillates were then dried over anhydrous sodium sulfate and concentrated to a volume of ~100 μl by means of Vigreux distillation and subsequent micro distillation (8). The distillates were stored at −80°C and analyzed within 4 weeks after the workup.

### Gas chromatography-olfactometry (GC-O)

For gas chromatography-olfactometry a Trace Ultra GC (Thermo Finnigan, Dreieich, Germany) equipped with either a DB-5 (30 m, 0.32 mm i.d., 0.25 μm film thickness, J&W Scientific, Fisons Instruments, Mainz-Kastel, Germany) or a DB-FFAP (30 m, 0.32 mm i.d., 0.25 μm film thickness, J&W Scientific, Fisons Instruments, Mainz-Kastel, Germany) capillary column was used. Samples were applied using the cold on-column technique (40°C). Therefore, 2 μl of the samples were manually injected on a pre-column (deactivated fused silica capillary, 3 m, 0.32 mm). When DB-5 columns were used, the initial temperature of 40°C was held for 5 min and was then raised with a rate of 8°C/min to 200°C. Thereafter, the temperature was raised by 15°C/min until the oven reached the final temperature of 300°C. This temperature was held for 5 min. When DB-FFAP columns were used, the initial temperature of 40°C was also held for 5 min and then raised with a rate of 8°C/min to the final temperature of 240°C. The final temperature was held for 5 min. Helium was used as carrier gas at a constant flow of 2.5 ml/min. To detect the odorous substances in the samples, the effluent was split after the analytical column by a glass Y-splitter and led to a flame ionization detector (FID) and a sniffing port using two deactivated fused silica capillaries (0.7 m, 0.32 mm). Both detectors were held at a temperature of 250°C. For identification, the odor as well as the retention indices (RIs) on two columns with different polarity were compared to those of reference substances. The retention indices were calculated by using a series of n-alkanes (C_6_–C_26_) as described previously (9).

### Odor extract dilution analysis (OEDA)

The determination of the most potent odor-active compounds in acrylic paint was carried out by ranking them according to their relative intensity via a modified comparative OEDA (10). The initial distillate was diluted stepwise 1+1 (v/v) with DCM and an aliquot of each dilution step was analyzed by means of GC-O. The analysis was started with the odor dilution step (FD) 65,536 followed by the preceding dilution (FD 32,768). If an odor was perceived during GC-O, the same odor-active region in the preceding dilution step was marked with its odor description if the same olfactory impression was observed in both dilutions. Sniffing an odorous region was stopped, when either an odor could be perceived in two consecutive dilution steps, or the peak exceeded a height that approximately equaled a concentration of 20-50 μg/ml. The flavor dilution factor of a given compound is referred to as the highest dilution step yielding clear olfactory recognition of the compound.

### Determination of odor thresholds values

The odor threshold value of 3-methyl-4-heptanone in air was determined by GC-O using (*E*)-2-decenal as an internal standard (11, 12). The panel consisted of six people (3 male, 3 female) in an age range of 21 to 33 years (median: 26 years). For determination of the odor threshold, 2 μl of every dilution step was analyzed in the GC-O system, with every experiment being conducted once. The measurements were performed on a capillary DB-5 column. The initial temperature of 40°C was held for 2 min and was then raised with a rate of 8°C/min to 300°C. The final temperature of 300°C was then held for 5 min. Helium was used as carrier gas at a constant flow of 2.5 ml/min. Detection was performed as described for GC-O analyses (cf. Gas-chromatography olfactometry). The purity was taken into account for the calculation of the odor threshold values.

### Gas chromatography-mass spectrometry (GC-MS)

A 7890 A GC-System (Agilent, Waldbronn Germany) equipped with either a DB-5 or DB-FFAP capillary column (30 m, 0.32 mm i.d., 0.25 μm film thickness) was used for gas chromatography-mass spectrometry analysis. A sample volume of 1 μl was automatically applied on a pre-column (deactivated fused silica capillary, 2–3 m, 0.32 mm) using the cold on-column technique. The injection in the Cooled Injection System CIS4 (Gerstel, Duisburg, Germany) was performed using a multi-purpose sampler (MPS, Gerstel, Duisburg, Germany). The temperature program was as follows: The initial temperature of 40°C was held for 5 min and was raised with a rate of 8°C/min thereafter. When using a DB-5 column, the final temperature of 300°C was held for 5 min, whereas the oven temperature in case of the DB-FFAP column was held at 240°C for 5 min. Helium was used as carrier gas at a constant flow of 1 ml/min. Mass spectra were generated using an Agilent 5975C MSD quadrupole mass spectrometer (Agilent, Waldbronn, Germany) in full scan mode (*m/z* = 30–350) in the electron ionization (EI) mode at an ionization energy of 70 eV. For identification, the mass spectra and retention indices of the unknown odorants were compared to those of reference substances analyzed under identical conditions. Analytes were classified as identified, if they showed a match that was >920, a maximum RI difference of five and were described with the same odor qualities in GC-O analyses when compared to the reference substance. If no standards were available, the NIST 14 database was used for identification. However, identification was taken as only tentative in cases where no reference substance was available but the achieved database match score was >920.

### Heart cut two dimensional gas chromatography-mass spectrometry/olfactometry (GC-GC-MS/O)

The two dimensional system consisted of two Agilent 7890B gas chromatographs coupled with an Agilent 5977B mass spectrometer (Agilent, Waldbronn, Germany). The first system was equipped with a DB-FFAP column (30 m, 0.32 mm i.d., 0.25 μm film thickness). A MPS applied 2 μl sample volume on a precolumn (deactivated fused silica capillary, 3 m, 0.32 mm i.d.) that was connected to the CIS 4 injection system. The initial temperature of 40°C was held for 2 min and then raised at a rate of 8°C/min to a final temperature of 240°C (5 min). The effluent was led to a multi column switching system (MCS, Gerstel), where it was directed using deactivated fused silica capillaries to both, a flame ionization detector (FID) and an olfactory detection port (ODP) or a cryogenic trap system (CTS, Gerstel). The CTS was connected to the second GC system, equipped with a DB-5 column (30 m, 0.25 mm i.d., 0.25 μm film thickness). The effluent was split using a Y-splitter and led to the MSD and ODP using deactivated fused silica capillaries. Helium was used as carrier gas for both systems at a constant flow of 2.5 ml/min for the first system, and 1 ml/min for the second system. The FID and ODPs were held at a temperature of 250°C and 270°C, respectively. The MSD was operated at full scan mode recording *m/z* from 35 to 400 with an ionization energy of 70 eV (EI mode). For identification we used the criteria described above.

### Compliance with ethical standards

The study was conducted in agreement with the Declaration of Helsinki. The study (registration number 180_16B) was approved by the Ethical Committee of the Medical Faculty, Friedrich-Alexander Universität Erlangen-Nürnberg. Informed consent was obtained from all subjects participating in the study.

## Results

### Odor profiles and sensory evaluation

During the first step of the sensory evaluation, the panelists agreed on eight odor qualities that were mentioned by at least 50% of the panelists. The orthonasal sensory analysis of the acrylic paints revealed, by consensus, the following odor attributes: citrus-like, fruity, rubber/plastic-like, mushroom-like, turpentine-like, geranium leaf-like/metallic, pungent, and alcohol-like.

**Figure 1:**
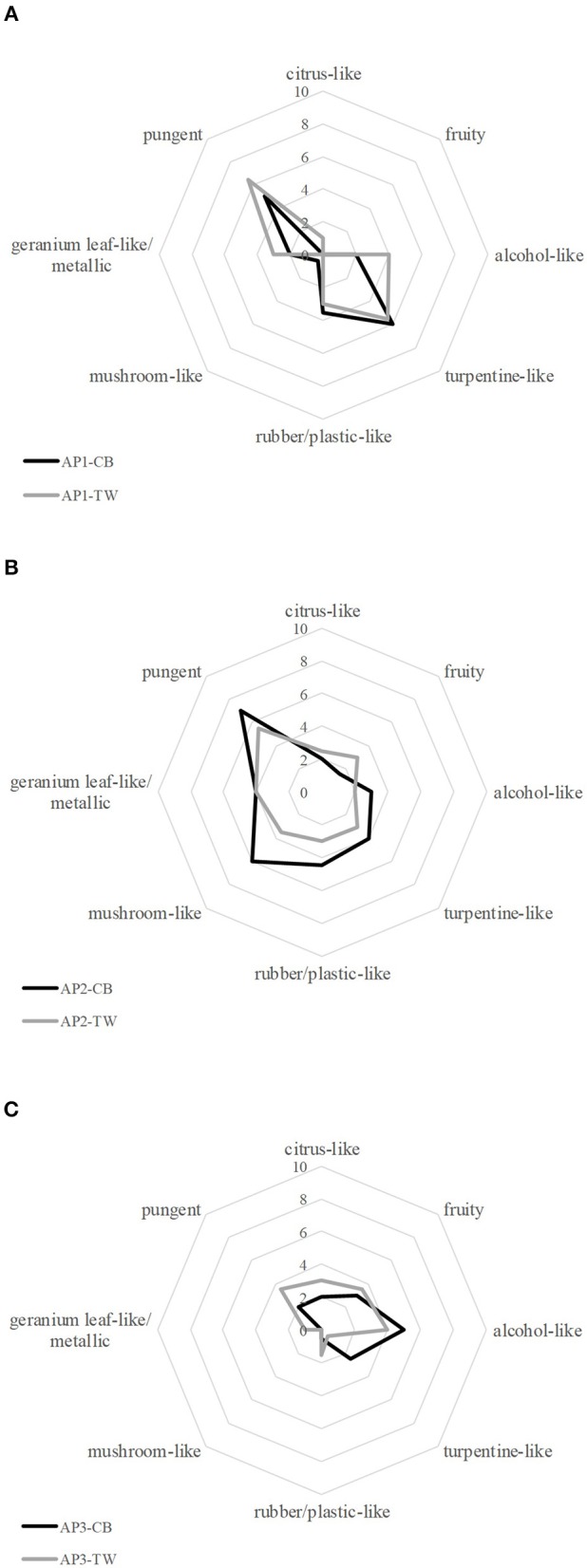
Comparative Odor profile of black (CB) and white (TW) acrylic paints (AP) from each manufacturer respectively [**(A)**: AP1, **(B)**: AP2, **(C)**: AP3].

Overall, acrylic paint (AP) samples obtained from different manufacturers revealed different dominant odor qualities and showed a great variety with regard to their overall odor intensity, the hedonic rating and the assessment of possible health hazards. However, paints from the same manufacturer showed similar results for the given characteristics (cf. Figure 1).

Both AP1 samples, black and white, showed similar odor properties with pungent and turpentine-like being the most abundant odor qualities represented by an average score of 5–6.5. The panel also rated the odor qualities rubber/plastic-like, alcohol-like and geranium leaf-like/metallic with a medium score of 2–3.5. Since the odor qualities mushroom-like, fruity and citrus-like were rated with a score of 0–1, all qualities that are associated with primarily positive odors were negligible in comparison to the remaining odor qualities. Alongside the strong perception of negative associated odors, a low hedonic score of 2.5 and 3 for the black and the white paint was recorded. Generally, subjects reported a rather repulsive and unpleasant odor. In relation to that, the subjective assessment of the potential health hazard was relatively high, with an average score of 8 for the black and 6.8 for the white color. The overall smell intensity was rated with 7 and 6.5, respectively, showing that both paints emitted comparably strong unpleasant smells.

Comparable results for both colors were also obtained for the sensory evaluation of the AP2 samples. Thereby, the pungent and mushroom-like odor impressions were rated highest with a score of 7 and 6, respectively for the black paint, and 5.5 and 3.5 for the white paint, respectively, whereas fruity, alcohol-like and citrus-like impressions were rated with low scores in the range from 1.5 to 3. The odor qualities turpentine-like, geranium leaf-like/metallic and rubber-/plastic-like were perceived with medium intensities in the range of 3 to 4.5. In the course of the hedonic evaluation, panelists reported the smell of the samples as rather unpleasant mirrored by a rating of 2 for the black and 3 for the white paint, being in line with the rating of their subjectively perceived potential health hazard (both 7), and the fact that both paints were reported to emit the strongest odor of all investigated samples, scoring 8 (CB) and 7.5 (TW) for the overall intensity. Moreover, the samples revealed a strong pungent odor.

The black and the white samples AP3 showed a higher divergence not only from the other samples but also between the black and the white sample with regard to their odor profile: the smell of the black paint was primarily described as alcohol-like, fruity, and turpentine-like, whereas the white paint revealed alcohol-like, fruity, pungent and citrus-like notes. The remaining odor qualities were either not perceived or rated with a low score by the panelists (≤ 1.5). The participants rated both samples as less intense than the other samples with an overall intensity rating of 5. Likewise, the smell of both samples was rated as less unpleasant, with a rating of 4 for both samples, and a lower assumed potential health risk with 4 and 4.5, respectively.

### Identification of odorous constituents

First, the volatiles were extracted with DCM followed by high vacuum distillation using the SAFE technique. The distillates obtained exhibited the characteristic overall smell of each kind of acrylic paint, proving the successful extraction of all key odorants. Next, the distillates were subjected to OEDA as a screening method to differentiate between the odor-active compounds and the bulk of odorless volatiles. Application of OEDA revealed a total of 36 compounds in the six analyzed AP samples in an FD factor range of 2 to 32,768. Thereof, 22 odorous substances were unequivocally identified by comparing their odor qualities, their retention indices (RI) on two columns of different polarity and their mass spectra to those of reference compounds. Further, four substances could be tentatively identified based on their odor and RI, as no mass spectrum was available due to their low concentrations in the samples or as there were no reference substances commercially available. If not mentioned differently, paints of the same manufacturer showed the same odor active compounds (cf. Table 2) and are therefore not presented separately.

**Table 2:** Chromatographic and organoleptic information of all identified substances and parameters of chemical identification.

			**RI on**		**FD factor**^**d**^					
**No.^a^**	**Compound^b^**	**Odor quality^c^**	**DB-FFAP**	**DB-5**	**AP1-TW**	**AP1-CB**	**AP2-TW**	**AP2-CB**	**AP3-TW**	**AP3-CB**
1	(*E*)-2-Butenal	Fruity, fermented	905	696	<1	<1	<1	<1	16	128
2	Butyl acetate	Fruity, pear-like, plastic-like	1,067	825	<1	<1	32	128	<1	<1
3	Ethylbenzene	Aromatic, gasoline-like, solvent-like	1,120	858	256	<1	32	64	<1	<1
4	3-Methyl-4-heptanone	Fruity, apple juice, hazelnut	1,142	931	512	512	2,048	4,096	128	32
5	1-Butanol	Fruity, alcohol-like	1,143	n.d.	<1	<1	256	1,024	<1	<1
6	Cumene	Aromatic, gasoline-like	1,166	929	1,024	512	1,024	1,024	<1	<1
7	Butyl acrylate	Geranium leaf-like, mushroom-like	1,175	903	64	512	32,768	65,536	32	16
8	Propylbenzene	Aromatic, fruity, solvent-like	1,196	957	2,048	128	256	512	<1	<1
9	sec-Butylbenzene	Aromatic, fruity, solvent-like	1,237	1,008	2,048	256	256	128	<1	<1
10	Styrene	Plastic-like, aromatic	1,249	892	128	256	128	256	<1	<1
11	Allylbenzene	Aromatic, solvent-like	1,250	947	<1	<1	64	16	<1	<1
12	Octanal	Citrus-like, soapy, fatty	1,279	1,007	<1	<1	<1	<1	128	8
13	2-Acetyl-1-pyrroline^e^	Roasty, popcorn-like	1,312	920	<1	<1	<1	<1	16	32
14	Methyl octanoate	Yeasty, fermented	1,378	1,129	<1	<1	64	128	<1	<1
15	Acetic acid	Vinegar-like	1,443	n.d.	<1	<1	4	<1	<1	2
16	Trimethylindane^*^	Mothball-like, plastic-like	1,460	n.a.	256	32	<1	<1	<1	<1
17	2-Ethylhexyl acrylate	Plastic-like, garlic-like, fruity	1,469	1,232	<1	<1	512	512	<1	<1
18	Trimethylindane^*^	Mothball-like, plastic-like	1,470	n.a.	512	512	<1	<1	<1	<1
19	Decanal	Waxy, flowery	1,490	1,209	<1	<1	<1	<1	32	64
20	Benzaldehyde	Bitter almond-like	1,511	957	<1	<1	64	32	<1	<1
21	Tetramethylindane^*^	Mothball-like, plastic-like	1,635	1,336	16,384	16,384	<1	<1	<1	<1
22	Dimethyltetralin^*^	Anise-like	1,701	n.a.	256	512	<1	<1	<1	<1
23	Naphthalene	Mothball-like, plastic-like	1,728	1,178	2,048	128	<1	64	<1	<1
24	2-Methylnaphthalene	Mothball-like, plastic-like	1,835	1,192	64	<1	<1	<1	<1	<1
25	1,7-Dimethylnaphthalene	Mothball-like, plastic-like	1,978	1,411	512	<1	<1	<1	<1	<1
26	1,2-Dimethylnaphthalene	Mothball-like, plastic-like	2,055	1,452	4,096	4,096	<1	<1	<1	<1

a *Odorants are consecutively numbered according to their retention indices on capillary DB-FFAP*.

b *Odorant was identified by comparison of its odor quality and intensity and retention indices on capillaries DB-FFAP and DB-5 as well as mass spectra (EI mode) with data of reference compounds*.

c *Odor quality as perceived at the odor detection port*.

d *Flavor dilution factor determined by OEDA on capillary DB-FFAP*.

e *No unequivocal mass spectrum (EI mode) was obtained; identification was based on remaining criteria provided in footnote b*.

*n.d.: Not detectable on given capillary column due to partial or total coelution of the substance together with the solvent (DCM)*.

*n.a.: Odorous area could not clearly be associated with a specific RI*.

* *Methylation pattern could not be resolved unequivocally*.

With a few exceptions, the odorous compounds found in the AP1 samples were described as mothball-like, anise-like, plastic-like, aromatic, solvent-like, or fruity. Our findings showed that these samples contained three different naphthalene derivatives, namely 1,2-dimethylnaphthalene, 1,7-dimethylnaphthalene and 2-methylnaphthalene, as well as naphthalene itself, that contributed to the odor profile of AP1 paints. To the best of our knowledge, dimethylated naphthalene derivatives were identified for the first time as odorous substances in acrylic paint in the frame of this study. GC-O analysis showed that especially 1,2-dimethylnaphthalene was able to contribute to a strong mothball- and plastic-like odor since it could be perceived at FD-factors up to 4,096 during OEDA. Likewise, naphthalene showed a plastic- and mothball-like odor at FD-factors of 2,048 for the white, and 128 for the black paint and therefore had a high impact to the overall odor of AP1 samples. Furthermore, constituents with aromatic, solvent-like, gasoline-like, and plastic-like smell were identified as odor-active benzene derivatives. With FD factors of 512 (TW) and 128 (CB) or higher, primarily sec-butylbenzene, propylbenzene, and cumene were able to affect the odor of both samples. Showing a FD factor of 512 in both samples, the fruity, apple juice-like and hazelnut-like 3-methyl-4-heptanone adds fruity nuances to the odor of both samples. The acrylic monomer butyl acrylate showed a mushroom-like and geranium leaf-like odor that could be perceived until FD 512 in the case of the black color and was therefore considered to contribute to the odor of the AP1 samples.

The substance with the highest FD factor (cf. Table 2) and therefore most important to the smell of AP1 samples could be tentatively identified as a tetramethylindane (no. **21**; mothball-like, plastic-like). Since no reference compound was available, the exact methylation pattern could not be elucidated. The NIST database showed good matches with trimethylated tetralines as well as tetramethylated indanes as both substance groups show similar fragmentation patterns in mass spectrometric detection. However, the comparison of the retention indices with 1,5,7-trimethyltetralin and values found in literature showed, that the unknown substance correlates better with the indane derivatives, and was therefore tentatively identified as a tetramethylated indane.

Two substances with mothball-like and plastic-like odor were tentatively identified as trimethylated indanes (no. 16, 21), since no reference substances were commercially available. Since only hydrocarbon fragments were detected and the base peak showed a mass-to-charge ratio of *m/z* = 160, it was concluded that the molecular formula of both substances was C_12_H_16_. A comparison with the NIST database showed high matches for both, dimethylated tetralins and trimethylated indanes, as the fragmentation of both substances is nearly identical. For DB-FFAP columns the unidentified substances showed retention indices of 1,470 and 1,460, respectively, and therefore elute earlier than the non-methylated tetralin with a RI of 1,497. This leads to the conclusion that both substances are not based on tetralin. As there is insufficient data available regarding the retention behavior of trimethylated and dimethylated indanes, it was not possible to directly compare retention indices. Alternatively, the retention behavior was analyzed via incremental analysis using the retention indices of methylated indane derivatives. Literature data showed a retention index of 1,365 for indane (13) and 1,408 for 1-methylindane (14) on a polar capillary column, so that the RI increase for one additional methyl group is about 37. When theoretically extrapolating this value, a calculated RI of a 3-fold methylated compound would correspond to a value of about 1,480. While keeping in mind that this is just a theoretical approximation, the estimated RI would fit to the obtained values for both unidentified compounds and together with their well matching MS spectra and odor quality would lead to the assumption that both substances might be trimethylated indanes.

Apart from that, a substance with an anise-like odor was tentatively identified to be a dimethylated tetralin. The mass spectrum showed the highest signal at *m/z* ratio of 160 and furthermore only hydrocarbon fragments so that a general structure with the molecular formula C_12_H_16_ was expected. A search in the NIST database showed a good match with dimethylated tetralins, with a methyl group located at position two. The retention indices of the commercially available reference substances 1,5-dimethyltetralin and 2,6-dimethyltetralin showed values of 1,714 and 1,650, respectively. The unknown substance revealed a retention index of 1,701 and was therefore within the range of the dimethylated derivatives. Furthermore, its specific anise-like odor resembled the smell of two structurally related dimethylated naphthalenes, namely 2,6- and 2,7-dimethylnaphthalene, leading to the assumption that the respective tetralin derivatives might elicit a similar odor impression. This led to the conclusion that the unidentified substance with anise-like odor might be a dimethylated tetralin.

The odorant with the highest FD factor (FD 65,536) in the AP2 samples, and therefore the strongest odorant of all samples, was found to be butyl acrylate (no. **7**; mushroom-like, geranium leaf-like). Additionally, a second acrylic monomer, 2-ethylhexyl acrylate (no. **17**), eliciting a plastic-like, fruity and garlic-like odor, could be identified in the AP2 samples. Since 2-ethylhexyl acrylate was only perceived until FD 512, its contribution to the odor of AP2 samples was considered as being relatively lower than that of butyl acrylate. The fruity 3-methyl-4-heptanone (no. **4**: fruity, apple juice-like and hazelnut-like) was perceived until FD 2,048 and 4,096 in the white and the black sample, respectively. Showing the second highest FD factors in these samples, 3-methyl-4-heptanone highly contributed to the fruity nuance of AP2 paints. Furthermore, a wide range of alkylated benzene derivatives with plastic-like, solvent-like and aromatic odors was detected with high sensory impact in both samples. Thereby, cumene (no. **6**) and propylbenzene (no. **8**) were most pronounced among these substances, revealing FD factors of 1,024 and 256, respectively, thereby eliciting aromatic, gasoline-like and solvent-like impressions. Nevertheless, no polycyclic aromatic hydrocarbons (PAHs) could be found in these samples except naphthalene (no. **23:** mothball-like, plastic-like), which was found in the black but not in the white paint. With a lower FD and therefore most likely with a lower influence on the odor of paint, acetic acid was identified in the white but not in the black color.

In agreement with the findings of the sensory evaluation of AP3, a smaller number of odorous molecules with generally lower FD values for all compounds was detected in AP3 samples. Screening via GC-MS also showed that AP3 did not contain any alkanes, benzene derivatives or PAHs and was therefore devoid of substances with mothball-like, aromatic or plastic-like odor. The fruity 3-methyl-4-heptanone (no. **4**: fruity, apple juice-like, and hazelnut-like) was identified to have the highest FD factors in the black and the white paint with factors of 32 and 128, respectively. Its odor threshold was determined as 0.032 ng/l_air_. Accordingly, we assume that the fruity odor that was observed in the sensory evaluation was likely to be caused by 3-methyl-4-heptanone. Apart from that, octanal (citrus-like, soapy, fatty) was found in both samples, but with divergent FD factors. Whereas, the black color showed a relatively low FD factor of 8, octanal could be perceived until FD 128 in the white color. As in the other samples, butyl acrylate could be additionally identified in AP3 samples as odorant with a geranium leaf-like and mushroom-like smell.

### Identification of non-odorous volatiles

Apart from the odorous substances that were determined in this study, a variety of non-odorous VOCs was identified via GC-MS (cf. Table 3). A high amount of n-alkanes, branched chain alkanes, cyclohexane and cyclopentane derivatives, as well as methylated benzene derivatives could be detected in AP1 samples. These samples already showed the highest content of odor active benzene derivatives and PAHs in previous tests and were also assessed with the highest rating for the odor qualities pungent and turpentine-like in the sensory evaluation. Furthermore, both samples did not only show a high variety and therefore a wide range of branched and cyclic alkanes, benzene derivatives and PAHs, but also contained high amounts of these substances in comparison to the odor-active constituents.

**Table 3:** Quantitatively dominating and potentially harmful VOCs that were identified by means of mass spectrometric detection.

	**AP1**	**AP2**	**AP3**
**ANTIFOAMING AGENT**			
Tributyl phosphate	x	x	
**CARRIER MATERIAL**			
1,2-Propanediol		x	
**COALESCING AGENT**			
1-(1,1-Dimethylethyl)-2-methyl-1,3-propanediyl diisobutyrate	x		
2,2,4-Trimethyl-1,3-pentanediol diisobutyrate		x	
2,2,4-Trimethyl-1,3-pentanediol monoisobutyrate (Texanol)		x	
2,2,4-Trimethyl-1,3-pentanediol		x	
**CONTAMINATIONS FROM MANUFACTURING PROCESS**			
1-Octanol			x
1-Decanol	x		
1-Dodecanol		x	
1-Tetradecanol		x	
Methyl palmitate	x	x	
Methyl linoleate	x	x	
Methyl oleoate	x		
**ODORLESS SUBSTANCES IN PAINT THINNER**			
n-Alkanes C8-C18	x		
Alkanes C8-C18, branched	x		
Mesitylene	x		
p-Cymene	x		
p-Xylene	x	x	
1,2,4-Trimethylbenzene	x		
Toluene	x		
Dimethylated cyclohexanes	x		
Trimethylated cyclohexanes	x		
Ethyl/ Methyl cyclopentane	x		
Ethyl/ Methyl cyclohexane	x		
**PLASTICIZER**			
Dimethyl phthalate	x		x
**PRESERVING AGENTS/BIOCIDES**			
4,4-Dimethyl-oxazolidine		x	
5-Chloro-2-methyl-3(2*H*)-isothiazolidine			x
**ADDITIONAL SOLVENTS**			
n-Dibutylether		x	
2-(2-Ethoxyethoxy) ethanol (Carbitol)		x	
2-(2-methoxyethoxy)ethanol			x

Coalescing agents could be identified in all analyzed paints except of AP3 samples. The used substances were based on a mono- or diester of 2-methylpropanoic acid and alkylated 1,3-alkandiols. Whereas, AP1 samples only contained small amounts of coalescing agents, AP2 samples showed high contents, so that the coalescing agent's peak area made up 81% of the total peak area of the whole chromatogram and therefore represented the main part of the VOC composition of these two samples.

Whereas, only AP2 samples contained the monomer butyl acrylate as a plasticizer in high amounts, dimethylphthalate was found as additive in the AP1 and AP3 samples. AP3 samples showed such a high concentration of dimethylphthalate that its peak area made up 83% of the total peak area, thus being the relevant constituent with regard to total VOC content.

Since acrylic paints are prone to microbial deterioration, they often contain biocides to improve shelf life of the products. Correspondingly, biocides could be identified via GC-MS in four of the analyzed samples. The AP3 samples that had been classified as “non-toxic” contained a common preserving agent for paints, namely 5-chloro-2-methyl-3(2*H*)-isothiazolinone. The biocide 4,4-dimethyl-oxazolidine was found as another additive in the AP2 samples.

Furthermore, the anti-foaming agent tributyl phosphate, the carrier material 1,2-propanediol and other contaminations that are not specified here in more detail and that most likely stemmed from the manufacturing process (Table 3) were identified in AP1 and AP2 samples.

## Discussion

### Sensory and hedonic evaluation

The odor profiles obtained in the sensory evaluation of AP2 samples correlated well with the evaluation of the hedonic rating and assessment of a subjectively perceived potential health risk for the black and the white paint. Thereby, the white sample, which was rated more positive in the hedonic evaluation, showed a lower fear of potential health hazards as well as a higher rating of positively associated odor qualities in the odor profile such as citrus-like and fruity. On the contrary, sensory analysis revealed similar odor profiles for both AP1 samples in the sensory evaluation despite the fact that the subjective rating of potential health hazards showed varying ratings between the black and the white paint. The fact that olfactory rating did not reveal such pronounced differences that would explain the differences regarding the potential hazard led to the conclusion that the divergence in rating of the potential health hazard might be due to visual cues rather than the olfactory percept elicited by the samples. Despite our efforts to minimize visual influence by using brown glass bottles, panelists might still have been influenced by the brightness of the contained paint in case of the white product, as the color white is often associated with purity or happiness in western countries (15). The positive association might then lead to a safer and healthier feeling even if the perceived odors differ only slightly. For AP3 samples the subjectively assumed potential health hazards, the hedonic rating and the overall intensity were evaluated as being nearly identical, so that differences in the sensory evaluation were mainly related to a divergence in perception of the odor qualities turpentine-like and alcohol-like. These were more intense in the black sample, whereas pungent and rubber-/plastic-like were more prominent in the white sample. However, differences in the evaluation of the odor qualities did not seem to have an impact on the assessment of health risks or the hedonic evaluation in this case. Despite the fact that different, negative connoted odor qualities were prominent in the respective sample, these qualities were rated with a similar intensity, leading to comparable olfactory impressions and associations. Summarizing the findings of the sensory and hedonic rating together with the subjective rating of a potential health risk, it can be concluded that a high rating of aversive impressions such as pungency and turpentine-like smells obviously goes along with a negative hedonic rating and higher subjective fear of potential health risks. However, a stronger perception of pleasant odor qualities such as citrus-like and fruity and lower intensity of the aforementioned aversive impressions correlated with lower fear of negative effects on the panelists' health.

### Characterization of odorous constituents

Comparison of the results of all GC-O analyses revealed that the majority of the identified odor-active substances originated from hydrophobic coalescing agents, paint thinners or added solvents; accordingly, the composition of these additives is highly relevant for the odor as well as a potential physiological hazard of acrylic paints. Depending on the thinner type, mineral spirits can contain up to 25% aromatic hydrocarbons and may therefore serve as an important source of odorous contaminants and potentially hazardous substances in paints. Amongst these constituents are benzene derivatives or PAHs, in particular naphthalene, indane and tetralin derivatives. Most of these compounds have been reported to be of toxicological relevance, namely ethylbenzene, (*E*)-2-butenal, allylbenzene, cumene, styrene and naphthalene (16–21). All in all, the impact on human health caused by the identified substances ranges from temporary dizziness to hepatic or nervous damages as well as carcinogenic effects (22–24). Other substances reported here, such as 3-methyl-4-heptanone, have not yet been investigated with regard to their potential physiological effects on humans. Our findings show that several of the substances contained in the paint samples could be perceived by smell, being described as gasoline-like, plastic-like, mothball-like, or aromatic. The sensory evaluation also showed that AP1 samples were rated as more intense with regards to odor attributes like turpentine-like and pungent, and also showed higher FD factors for these substances, demonstrating a clear correlation between the sensory evaluation and the results of the OEDA. Accordingly, AP3 samples that were devoid of benzene derivatives and PAHs, and showed no turpentine-like or pungent odorants during OEDA, were likewise rated lower with regards to these odor qualities. Consequently, the olfactory impression may serve as a hint with regards to problematic constituents and thus potential physiological harm; further studies would need to substantiate these observations.

The polymer dispersion that was present in all six samples was based on acrylic polymers; accordingly, acrylate monomers were identified in all paints investigated in this study. Thereby, 2-ethylhexyl acrylate could only be detected in two of the six samples, whereas butyl acrylate was found in all samples and was found to be obviously of high relevance for the smell of these acrylic paints; when being present in elevated concentrations, this substance exerts a characteristic mushroom-like and geranium leaf-like smell. Interestingly, the smell showed high resemblance to oct-1-en-3-one, a characteristic mushroom-like smelling substance that shows structural similarity to butyl acrylate with only one carbon moiety being replaced by oxygen. Overall, variable levels of butyl acrylate were observed in the samples, correlating to variable smell impressions in relation to the mushroom-like and geranium leaf-like note that might be linked to the following possible reasons: butyl acrylate either might have been added intentionally as a plasticizer, or may be a contaminant originating from impure acrylic polymer dispersions. In case of the AP2 samples, both paints were rated with the highest intensities regarding the odor quality mushroom-like/metallic by the panelists, being in line with the fact that butyl acrylate was by far the most potent odorant in the OEDA reaching FD factors of 32,768 and 65,536 in the white and the black paint, respectively. In case of AP1 and 3, this substance was only perceivable until FD 512. Since butyl acrylate was, however, found in all analyzed samples with high FD factors, this substance appears to be one of the main targets that would need to be reduced in concentration when aiming at developing odorless paints. Thus, the odor reduction could be accomplished by replacing butyl acrylate with an odorless alternative as plasticizer or aiming at using uncontaminated polymer dispersions.

Apart from that, 3-methyl-4-heptanone was detected in all analyzed samples as additional odorous substance. This substance has previously been reported in a wide range of food-related materials, namely hazelnut (25), coriander leafs (26), or olive oil (27). This substance is described here as a constituent of acrylic paints for the first time. Since 2,6-dimethylheptan-4-one is known to be a common solvent for varnishes and coatings, 3-methyl-4-heptanone might have been introduced into the paint as a by-product of 2,6-dimethylheptan-4-one. 3-Methyl-4-heptanone is not known to have any acute or chronic toxicological effects on humans, and is generally reported with a pleasant odor. Moreover, only low concentrations of this substance were found in all six samples. Nevertheless, one should keep in mind the relatively high odor impact of this substance with an odor threshold of 0.032 ng/l_air_.

Acetic acid is a common degradation product of acetic acid-derived esters such as butyl acetate, and may further stem from microbial degradation processes; accordingly, acetic acid might provide some information about a product's quality status. With regard to the investigated paints, AP2 white paint und AP3 black plaint showed elevated higher concentration of acetic acid than their corresponding samples from the same manufacturer; the reasons for this, however, have not been clarified in this study.

Apart from that, odor active aldehydes were detected yielding the highest OEDA values in the AP3 samples, with the citrus-like octanal and the fruity (*E*)-2-butenal that have been detected with the highest FD factors in the black and the white paint, respectively. This observation corresponds with the high rating of fruity and citrus-like notes in the sensory evaluation. The aldehydes octanal, decanal and (*E*)-2-butenal are common fat oxidation products being generated from unsaturated fatty acids. Residual fatty acids and their oxidation products have been previously reported in diverse materials, namely lubricants and packaging material, to name but a few (28, 29). Their presence in the investigated paints would need to be traced back by additional investigations on the single raw materials, however, lubricants and migration from the packaging can be assumed as likely sources.

When comparing the findings of the OEDA with regard to differences between white and black paints from the same manufacturer, it is apparent that the influence of the pigments is negligible in AP1 and AP3 samples. However, in case of AP2, naphthalene was identified as potent odorant in the black paint but not in the white one. Apart from its occurrence in paint thinners and solvents, naphthalene is also known to be a contaminant in black pigments called carbon black. Since the pigment “lamp black” is usually produced by incomplete combustion of heavy petroleum products like tar or by collecting soot, the pigments might contain contaminations originating from the raw materials and therefore comprise PAHs like naphthalene and its derivatives. Since naphthalene was only found in the black but not the white color, it is likely that the contamination was caused by such contaminated pigments used in the manufacturing process.

### Odorless volatile compounds

Nevertheless, elevated levels of non-odorous volatile substances may be a potential risk for the health of painters. It is noteworthy that a multitude of alkanes and hydrocarbons, most likely originating from paint thinners, was prevalent in the AP1 samples. Such compounds have already been reported with negative effects to the human health (22). AP2 and AP3 samples, on the other hand, contained less hazardous volatile substances such as 1-butanol or methylcarbitol or carbitol.

Nevertheless, the results of the GC-MS analyses demonstrated that AP3 samples primarily contained the plasticizer dimethyl phthalate (DMP). Studies showed that phthalates containing C_3_-C_5_ alkyl side chain may have an influence on testosterone biosynthesis, semen quality and human obesity as they can act as hormone-like substances (30–33), whereas DMP has not been reported with a negative effect on humans. However, its metabolite monomethyl phthalate (MMP) was found to diminish semen activity in humans (34) and might have an adverse effect on the growth of children (33).

Since solvent-based paints are nowadays replaced by water-based paints, preservatives need to be added to extend the shelf life of these paints. The biocides 4,4-dimethyl-oxazolidine and 5-chloro-2-methyl-3(2*H*)-isothiazolinone (CMIT), which could be identified in AP2 and AP3, respectively, are commonly used preservatives in paints and varnishes. CMIT is mostly administered as a mixture with methylisothiazolinone (MIT) (35) and is, however, known to cause dermal allergic reactions in 0.4–11.1% of the population of different European countries at skin contact (36). To prevent sensitization and allergic reactions to the paints, skin contact should be reduced to a minimum, accordingly.

## Conclusion

In this study we identified the substances that are responsible for the odor of fresh acrylic paint for artists. The turpentine-like, pungent or mushroom-like odor that was described by the panelists in the sensory evaluation could be traced back to a number of benzene derivatives, PAHs and acrylate monomers that either originate from paint thinners, from acrylic polymer dispersions or are added as a plasticizer. In this context the grade of the used paint thinner is of high importance. The higher the purity of the paint thinner and the lower the content of benzene derivatives and PAHs, the lower are the odor emission and the likely associated health risk. This is especially valid to be considered in view of the fact that these substances are either known to cause hepatic or nervous damage or have been reported as potential carcinogens. In addition, the choice of plasticizer was shown to be of importance for the odor of the analyzed samples. Whereas, paints with a high content of acrylate monomers, especially butyl acrylate, showed a noticeable mushroom-like and geranium leaf-like odor, the samples that were manufactured using different, odorless plasticizers were rated lower regarding these odor qualities. To reduce the unpleasant odor in acrylic paint and to minimize the potential risk of negative physiological effects on humans, the reduction of benzene derivatives, PAHs and acrylic monomers is advisable.

The results further indicate that the smell of fresh acrylic paint is not generally influenced by the pigments only, and does not necessarily depend on the color of the paint. However, there was one case of a black pigment that contained a divergent odor profile and elevated levels of PAHs that was most likely linked to usage of a Carbon Black pigment. Accordingly, this aspect will require more attention in future studies, and, consequently, stricter quality control. Substitution of insufficiently purified additives, such as pigments, turns out to be a valid strategy to reduce contaminants such as PAHs, thereby reducing potential health risks as well as smell nuisances in paints.

## Author contributions

Each author has participated in the work intellectually or practically, to take public responsibility for the content of this article, including the conception, design and conduct of the experiment and data analysis, as well as interpretation. PB carried out the practical work and data analysis. PB and AB conceived and designed the study, interpreted the results and contributed to the manuscript. The final version was approved by all authors.

### Conflict of interest statement

The authors declare that the research was conducted in the absence of any commercial or financial relationships that could be construed as a potential conflict of interest.
